# Skills for future equine sports rehabilitation careers

**DOI:** 10.1093/tas/txad042

**Published:** 2023-04-24

**Authors:** Sarah A Reed, Lisa N Streff

**Affiliations:** Dept of Animal Science, University of Connecticut, Storrs, CT 06269, USA; Dept of Animal Science, University of Connecticut, Storrs, CT 06269, USA

**Keywords:** curriculum, employer, equine, skills, sports rehabilitation, undergraduate education

## Abstract

The field of equine sports medicine and rehabilitation provides a career opportunity for students interested in remaining in the horse industry but not focused on a career as a veterinarian. However, throughout the United States, there are limited educational opportunities for undergraduate students to prepare for this career. The objective of this work was to determine what skills and theoretical knowledge professionals in the equine rehabilitation industry deemed most useful for employment in the equine rehabilitation industry, and, using that information, develop a curriculum to meet these industry needs. To meet this objective, a Qualtrics survey was distributed through email and social media to veterinarians, veterinary professionals, rehabilitation service providers, and horse owners. In addition to demographics, the survey asked respondents to list practical skills and theoretical knowledge that are essential for professionals in the equine rehabilitation industry. The majority of the 117 respondents (84%) were located in the United States, with the remainder from Canada (5%), the United Kingdom (5%), and several other countries. Eighteen percent of respondents were veterinarians, 26% owned or managed rehabilitation facilities, 8.5% were veterinary technicians, and the remainder were horse owners, rehabilitation service providers, and others. Horse handling skills (19%) and communication skills (18%) were the most commonly listed practical skills deemed essential for rehabilitation professionals. Of the theoretical skills, evaluation of lameness (29.5%), anatomy (31%), and fundamentals of equine reconditioning programs (32%) were deemed equally important for rehabilitation professionals. These data were used to design a minor in Equine Sports Rehabilitation that incorporated fundamental knowledge in lameness evaluation and rehabilitation methods as well as significant hands-on opportunities with rehabilitating horses and communicating about rehabilitation methods and progress with clients.

## INTRODUCTION

Experiential learning opportunities in undergraduate curriculums are recognized as a primary driver of success in postgraduation career placement (Robinson and Mulvaney, 2018; Crawford and Fink, 2020). Animal Science programs are generally designed to provide foundational scientific knowledge coupled with practical hands-on experience (Buchanan, 2008). These experiential learning opportunities come in many forms including lab-based coursework, internships, and undergraduate research and teaching opportunities. Employers of Animal Science graduates desire students to have knowledge in basic animal science, work experience relevant to the position, a strong work ethic, and communication skills, among other desired knowledge, experience, and competencies (Robinson and Mulvaney, 2018). Competence in disciplinary and nontechnical skills can be gained through experiential learning. Equine Science programs have the additional benefit of increasing satisfaction with a student’s educational experience and improving retention in Animal Science programs (Wood et al., 2010).

The horse industry itself is responsible for US$55 billion dollars of direct input to the United States GDP (American Horse Council, 2017). Directly employing almost 1 million people (Council, 2017), it is an avenue for career placement for undergraduate students. However, this industry, like many other animal science-related industries, requires significant practical experience. In particular, the field of equine sports rehabilitation is a growing niche within the industry (Kaneps, 2016; McGowan and Cottriall, 2016) but requires a specific skill set beyond what is generally taught in most baccalaureate programs. Employers have also called for a better incorporation of “transferable skills” into curriculum, with intentional placement (Easterly et al., 2017). Rehabilitation facilities provide services to owners who do not have the time, expertise, or facilities to properly rehabilitate an injured animal. However, in the United States, there is no clear degree or training path for nonveterinarians interested in equine rehabilitation, and, although regulations by state vary, no national certification or licensing is required. Thus, the objective of this work was to determine what skills and theoretical knowledge professionals in the equine rehabilitation industry deemed most useful for employment in the equine rehabilitation industry, and, using that information, develop a curriculum to meet these industry needs.

## MATERIALS AND METHODS

The procedures used in this study were approved by the University of Connecticut Internal Review Board, protocol X21-0095. The survey was designed in consultation with equine rehabilitation experts and veterinarians. The eight-item questionnaire included demographic questions and topical questions related to practical skills and theoretical knowledge required for lay professionals in the equine rehabilitation industry. The questionnaire was entered into Qualtrics for distribution (Supplement 1). The survey was distributed electronically through social media accounts, the University of Connecticut Equine Extension and the American Farrier’s Association listservs, direct email to equine rehabilitation facilities with a presence on the internet, and equine trade publications. Additional participants were recruited via word of mouth. No follow-up interviews were conducted. Partial survey responses were removed prior to data analysis. Descriptive statistics were run for each variable. Data are presented as percent of responses. Total percent may be greater than 100% for questions where multiple responses could be selected. Open ended questions were assigned topics using the Text iQ function in Qualtrics, allowing categorization into parent topics and analysis of frequency.

## RESULTS

### Demographics

**Figure 1.**
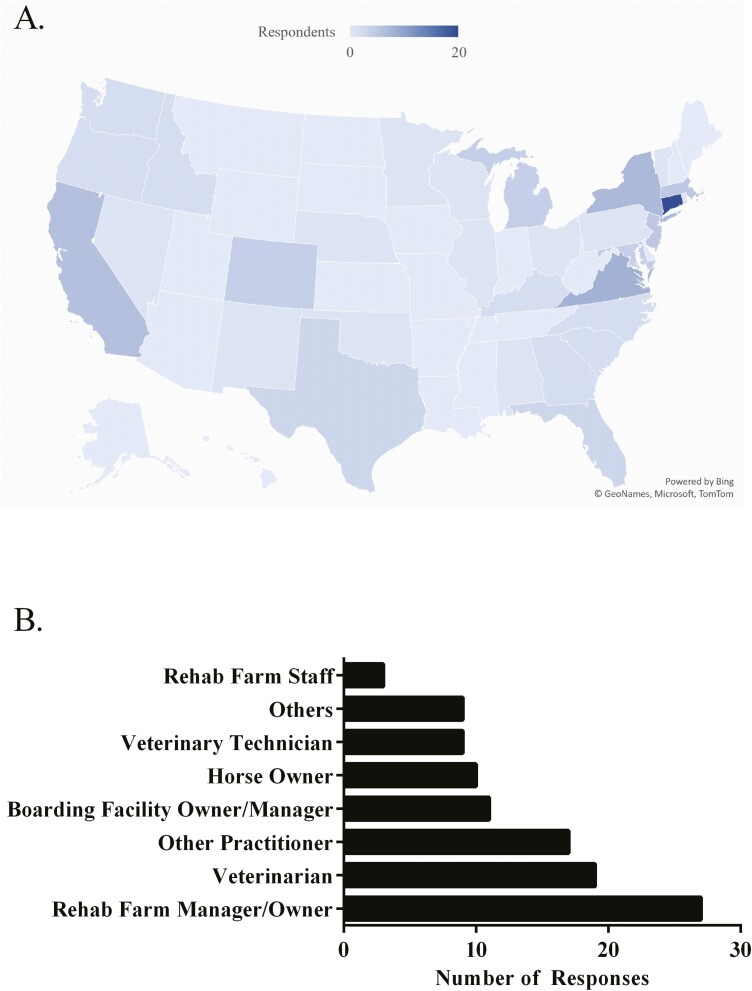
Demographics of respondents. Of 98 responses from the United States, 46.3% were located within the Northeast, 25.3% were located in Southern states, 18.9% were located in Western states, and 9.5% were located in Midwestern states. Of all 117 respondents, most were rehabilitation farm owners or managers, veterinarians, or other practitioners.

A total of 117 responses were returned from the survey (Figure 1A). Of those, 84% (*N* = 98 responses) were located within the United States. Canada (5%; 6 responses) and the United Kingdom (5%; *N* = 6 responses) were also represented within the respondents. Remaining respondents were from Australia, France, Ireland, Lithuania, New Zealand, Spain, and Switzerland (*N* = 1 respondent each). Of the respondents from the United States, the majority (46.3%; *N* = 44) were located within the Northeast (Connecticut, Delaware, Maine, Maryland, Massachusetts, New Hampshire, New Jersey, New York, Pennsylvania, Rhode Island, Vermont, and West Virginia), 25.3% were located in Southern states (*N* = 24 responses; Alabama, Arkansas, Florida, Georgia, Kentucky, Louisiana, Mississippi, North Carolina, Oklahoma, South Carolina, Tennessee, Texas, Virginia), 18.9% were located in Western states (*N* = 18 responses; Alaska, Arizona, California, Colorado, Hawaii, Idaho, Montana, Nevada, New Mexico, Oregon, Utah, Washington, Wyoming) and 9.5% were located in Midwestern states (*N* = 9 responses; Illinois, Indiana, Iowa, Kansas, Michigan, Minnesota, Missouri, Nebraska, North Dakota, Ohio, South Dakota, Wisconsin). Of respondents, most were rehabilitation farm owners or managers (*N* = 27; Figure 1B), veterinarians (*N* = 19), or other practitioners (*N* = 17).

### Practical Skills

**Figure 2.**
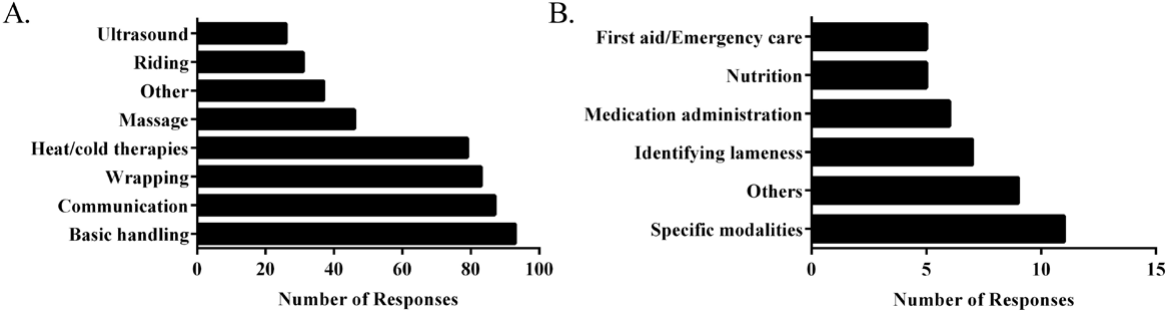
Practical skills identified by survey respondents as important. Responses to the question “What practical skills do you believe are essential for professionals in the equine rehabilitation industry?” (A) and categorization of “other” responses (B).

In response to the question “What practical skills do you believe are essential for professionals in the equine rehabilitation industry?”, basic handling was the most selected response (*N* = 93 responses, Figure 2A). This was closely followed by communication (*N* = 87 responses), wrapping (*N* = 83 responses), and hot and cold therapies (*N* = 79 responses). “Other” responses accounted for 37 total responses. When evaluated, these responses were further categorized (Figure 2B) into knowledge of specific treatment modalities (*N* = 11), identifying lameness (*N* = 7), administering medication (*N* = 6), nutrition (*N* = 5), and first aid/emergency care (*N* = 5), with nine other responses that did not fit in to other categories. Basic handling (32%) and communication (24%) were the most frequent skills listed as “most important” by respondents.

### Medical Knowledge

**Figure 3.**
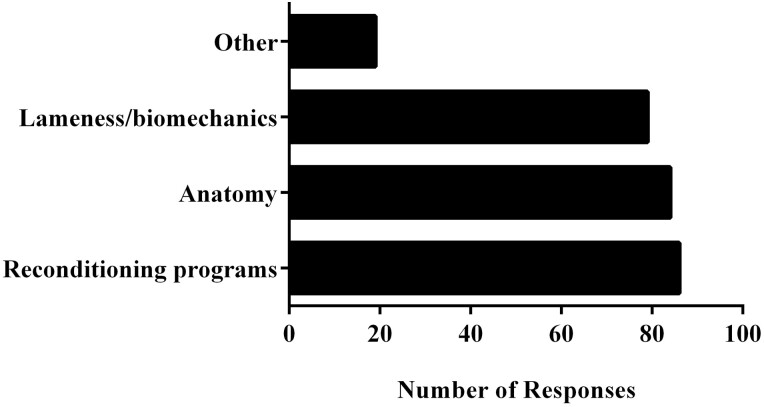
Medical knowledge identified by survey respondents as important. Responses to the question “What medical knowledge do you believe are essential for professionals in the equine rehabilitation industry?”

In response to the question “What medical knowledge do you believe are essential for professionals in the equine ­rehabilitation industry?”, respondents identified fundamentals of exercise reconditioning programs (*N* = 86 responses), anatomy (*N* = 84 responses), and evaluation of lameness/biomechanics (*N* = 79 responses) as top choices (Figure 3). “Other” choices (*N* = 19) included knowledge of first aid and emergency care (*N* = 9), physiology and behavior (*N* = 4), specific modalities (*N* = 3), and critical evaluation of medical data/use of modalities (*N* = 2). Fundamentals of exercise reconditioning programs (35%) and anatomy (31%) were identified as the two “most important” areas of knowledge by respondents.

### Desired Characteristics of Potential Employees

**Figure 4.**
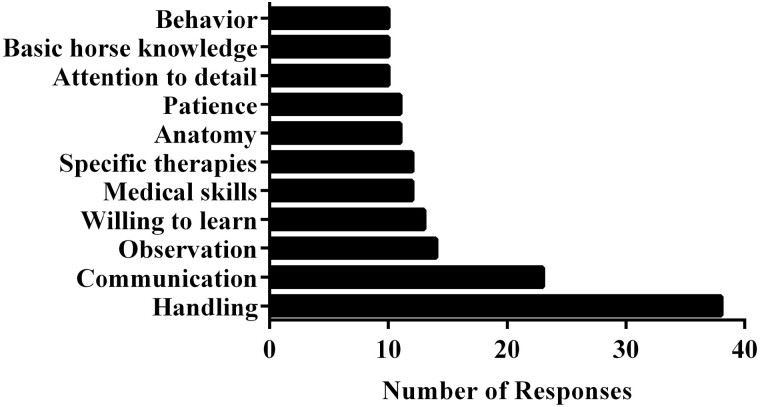
Top competencies desired by potential employees. Desired competencies of potential employees, indicated by survey respondents.

There were 65 responses to the question “Are there specific skills or qualities you look for in employees that will be rehabilitating horses? If so, what are they?” Two were excluded (“N/A”). Within Qualtrics, responses were tagged with key words, and which were then sorted in to three parent topics: Transferable skills (36% of responses), practical skills (34% of responses), and knowledge (28% of responses; Table 1). Handling skills (14% of responses) were the top desired skill of potential employees, with communication (9%) and observation (5%) ranked second and third, respectively (Figure 4).

**Table 1.** Parent topics and associated tags^1^

Knowledge	Practical skills	Transferable skills
Anatomy Basic horse knowledge and management Behavior Common sense Human rehabilitation Identify lameness Intelligence Nutrition Progression Scientific literacy Specific modalities	Bandaging Groundwork Handling Medical skills Record keeping Reporting Riding Situational awareness Wrapping	Attention to detail Calm Communication Critical thinking Determined Empathic Flexible Follows instructions Goal setting Observant Passionate Patient Professional Punctual Shows up Takes initiative Teamwork Willing to learn Work ethic Works independently

^1^Within Qualtrics, responses to “Are there specific skills or qualities you look for in employees that will be rehabilitating horses? If so, what are they?” were tagged with key words and sorted in to three parent topics: transferable skills, practical skills, and knowledge.

### Development of Learning Outcomes and Minor Plan of Study Curriculum

Based on the data collected through the survey, learning objectives for a new minor plan of study were developed. These learning objectives were designed to meet the primary demands of the practitioners in the industry, namely hands-on experience, knowledge of anatomy and physiology, critical thinking, and appropriate “transferable skills.” The learning objectives are shown in Table 2. Existing courses in Animal Science, Kinesiology, Allied Health, Agricultural Resources and Economics, and Nutritional Sciences were leveraged to meet these objectives. Curriculum mapping is provided in Table 3. Courses providing fundamental knowledge in exercise physiology and rehabilitation modalities, experiential learning opportunities in groundwork and rehabilitating horses, and business management are included within the required courses. Elective courses include opportunities to gain more in-depth knowledge in several areas.

**Table 2.** Equine sports rehabilitation minor learning objectives^1^

Learning objectives
1. Describe how equine anatomy and physiology impacts exercise.
2. Describe the benefits and limitations of different rehabilitation therapies.
3. Evaluate scientific evidence for or against using a therapy, integrating data from multiple sources and species.
4. Demonstrate proficiency at handling horses on the ground including fractious horses and those with behavioral challenges.
5. Demonstrate effective work in a team setting, with positive interactions and effective communication skills allowing communication with veterinarians, farriers, clients, and others involved in an animal’s rehabilitation.
6. Demonstrate effective management skills, including commitment to the rehabilitation plan, patience, and record keeping.

^1^Based on the data collected through the survey, learning objectives for a new minor plan of study were developed.

**Table 3.** Curriculum map for equine sports rehabilitation minor^1^

Course number	Course name	Learning objectives
“Required courses”		
ANSC 3311	Comparative exercise physiology	1, 2, 3
“or”		
KINS 4500	Physiological systems in human performance	1, 2, 3
ANSC 3551	Equine training I—groundwork	4, 5
ANSC 3554	Equine sports rehabilitation practicum	4, 5, 6
ANSC 3555	Equine sports rehabilitation seminar	2, 3
ANSC 3691	Professional internship	4, 5, 6
ARE 2210	Essentials of accounting and business	6
“or”		
ARE 2215	Business management	6
“Elective courses (must take at least one)”		
KINS 3522	Biomechanics of injury and sport	1, 2, 3
KINS 4510	Mechanisms and adaptations in sport and exercise	1, 2, 3
NUSC 4250	Nutrition for exercise and sport	3

^1^Courses included in the equine sports rehabilitation minor are mapped to the learning objectives they meet (see Table 2).

## DISCUSSION

Providing experiential learning activities for students that lead to development of career readiness competencies is a critical part of education in many animal science programs. Here, we describe the critical skills and knowledge required for employment in the equine rehabilitation industry, as identified by professionals in that industry.

### Practical Skills

Rehabilitation is generally categorized into the acute phase, which often requires significant veterinary attention and work with other service/healthcare providers (e.g., farrier, physiotherapist), and the “return to work” phase, which is a longer phase where the animal slowly gains fitness, while minimizing the risk of reinjury. Both phases of rehabilitation require specific practical skills and training. Given that the majority of time (likely more than 50%) in an equine rehabilitation position will be spent working with individual horses, it is not surprising that basic equine handling was identified as the most selected skill required and thus is incorporated into learning objective 4. In fact, “controlled hand walking” was the most frequently used modality of rehabilitation in a global study of rehabilitations specialists (Wilson et al., 2018). To meet this demand, students earning the minor are required to complete equine training I—groundwork, equine sports rehabilitation practicum, and a professional internship. The equine sports rehabilitation practicum requires students to work with an assigned horse for 4 d per week to meet the horse’s rehabilitation goals, under supervision of and with support from a faculty member. The professional internship is designed to provide students with hands-on opportunities using modalities that are not yet available in the University of Connecticut Equine Program (e.g., water treadmill). In equine training I, students work with weanling and yearling horses, developing handling and groundwork skills. In particular, because these are young, untrained horses, students gain experience and confidence working with horses that sometimes have unpredictable behavior. The handling skills identified as important in this survey are similar to those identified in Gadd et al. (2018), where equine industry professionals also listed basic horse handling and management as critical for employment. Moreover, in both studies, riding and training were relatively low on the required list of competencies desired by employers. This is important, as some students may not feel this is a valid career option because of a lack (real or perceived) of riding skill.

Further, due to the frequent interactions with other caregivers (veterinarians, farriers, others), it was unsurprising that communication was also high on the list of required skills. The importance of the relationship (i.e., communication) between different care providers has been previously identified (Moyer et al., 2012). Learning objective 5, “Demonstrate effective work in a team setting, with positive interactions and effective communication skills allowing communication with veterinarians, farriers, clients, and others involved in an animal’s rehabilitation” was designed to meet this need. In the equine sports rehabilitation practicum, students are required to communicate with the instructor as “the client” or owner of the horse, providing progress updates. Students also interact with the veterinarian and farrier, providing additional opportunities to practice professional communication. In the professional internship, it is expected that students will communicate with veterinarians, clients/owners, and other care takers. Other courses in the minor also incorporate forms of verbal and written communication, allowing for additional learning opportunities.

### Medical Knowledge

It is not surprising that fundamentals of exercise reconditioning programs, anatomy, and evaluation of lameness/biomechanics were identified as top responses to “What medical knowledge do you believe are essential for professionals in the equine rehabilitation industry?” These responses are incorporated into learning objectives 1, 2, and 3. To meet the learning objectives, students are required to take an upper (3000 or 4000) level exercise physiology course in Animal Science or Kinesiology, Equine Sports Rehabilitation Seminar, Equine Sports Rehabilitation Practicum, and can opt to take additional kinesiology courses. The exercise physiology courses both have prerequisite anatomy and physiology courses, ensuring students have fundamentals of anatomy and physiology. Fundamentals of exercise programs are covered in both exercise physiology courses, and both require evaluation of scientific evidence. The Equine Sports Rehabilitation Practicum incorporates lameness evaluations as students assess the progress of their horse. Other responses to this question included knowledge of first aid and emergency care, behavior, specific modalities, and critical evaluation of medical data/use of modalities. These are addressed in the Equine Sports Rehabilitation Practicum and Seminar courses. In particular, the seminar covers specific modalities through discussions and demonstrations, including the mechanisms and when each should or should not be used. The practicum includes recognition of stress and pain behaviors, how to handle horses with challenging behaviors, and first aid. Further, these courses/experiences will provide opportunities to learn and practice bandaging and wrapping, administering medications (under supervision of the instructor), record keeping and reporting (through required assignments in the Practicum), and situational awareness as students continually evaluate the horses they are working with. It is expected that the professional internship will also address these learning objectives by providing additional situations to apply the learned knowledge.

In response to the question “Are there specific skills or qualities you look for in employees that will be rehabilitating horses? If so, what are they?”, potential employers focused on three general areas—transferrable skills, practical skills, and knowledge. Transferable skills are defined as those that can be used in multiple situations or careers (Whalley, 2009), including skills and traits such as attention to detail, flexibility, patience, willingness to learn, and strong work ethic. These skills are incorporated into the hands-on courses, as students work directly with young and rehabilitating animals. These skills have been recognized as important in many careers, in fact, the National Association of Colleges and Employers (NACE) includes communication, work ethic, teamwork, and critical thinking as four of eight core competencies that employers seek in job applicants (National Association of Colleges and Employers, 2022). A 2020 Association of Public and Land-Grant Universities survey identified “Communicate accurately and concisely”, “Transfer knowledge from one situation to another”, and “Accept and apply critique and direction in the workplace” as three skills that had the largest gap between importance to employers, alumni, current students, and faculty, but the least amount of preparedness (Crawford and Fink, 2020). Others have demonstrated improvements in students’ assessments of their own patience, confidence, verbal communication skills, and teamwork following equine behavior and handling classes (Evans et al., 2009). Thus, the equine sports rehabilitation practicum and equine training I—groundwork can be used to gain transferable skills. Further, employers prefer students who have practical experience and look for work or internships on student resumes to build skills important for employability more than other activities (e.g., organizations, volunteerism, research, or travel; Robinson and Mulvaney, 2018; Crawford and Fink, 2020).

Survey respondents identified several practical skills as desirable in potential employees. Many of these skills are related to basic horsemanship and handling, with few listing riding as a desired skill. In a study of rehabilitation providers, it was identified that lay people (nonveterinarians), trainers, and owners were frequent providers of hand walking, massage, treadmill, hot and cold therapies, and bandaging (Wilson et al., 2018), similar to the expectation of rehabilitation providers in our study. The ability to handle many different types of animals and work with and rehabilitate horses from the ground (not riding) were also identified as important skills. Further, record keeping and reporting were also identified, in line with the desire for excellent communication skills that were identified in earlier questions. These skills allow rehab professionals to effectively care for injured horses, communicate with veterinarians, owners, and other practitioners, and document progression or lack thereof. The hands-on courses incorporate these skills into daily and weekly coursework, as students document and communicate changes in their horse’s progress over the semester.

Behavior, identifying lameness, and basic horse knowledge and management were among the “knowledge” characteristics identified as desired in potential employees. These skills and knowledge are incorporated into equine training 1—groundwork, equine sports rehabilitation practicum, and the professional internship. Employers also valued knowledge of anatomy and nutrition, which is included in the exercise physiology courses, as well as potential electives (e.g., nutrition for exercise and sport). Further, scientific literacy was also identified as a desired characteristic. Scientific literacy includes content knowledge, scientific practices, identifying and judging scientific expertise, and application of science to real-world scenarios (National Academies of Sciences, 2016). These skills are incorporated into several courses, including the exercise physiology courses and Equine Sports Rehabilitation Seminar, in which students use primary scientific literature to support or refute claims about exercise or therapeutic modalities.

Learning objectives for Animal Science curricula generally cover nontechnical skills such as critical thinking, scientific communication, and scientific literacy in addition to discipline specific competencies such as knowledge of domestic animal husbandry and management, physiological processes underlying the development and production of food and other domestic animals, and the structure and function of the animal food and fiber industry (Robinson and Mulvaney, 2018). Many animal science programs incorporate experiential learning to meet these learning objectives (Erickson, 2020). Species- and discipline-specific experiences can improve student preparedness for careers in animal science. The creation of specific minor plans of study within the broader animal science curriculum provides opportunities for students to gain more in-depth experience within their area of interest. For example, students enrolled in the equine sports rehabilitation minor are required to participate in a sports rehabilitation internship. This provides additional experiential learning opportunities, meeting general animal science curriculum and specific equine sports rehabilitation minor learning objectives. The minor integrates with the general animal science curriculum, as several courses in the minor can also be used to fulfill requirements for the bachelor’s degree.

Limitations to this study include a lack of available data on industry demand for positions in equine sports rehabilitation. In the United States, there is no national governing body that tracks individuals in equine sports medicine outside of the veterinary field, and requirements for rehabilitation practitioners vary from state to state. Thus, our understanding of the job market and demographics of practitioners is limited. Of note, several respondents noted that in their areas, there was a shortage of facilities and individuals willing to do rehabilitation work. However, additional research is needed to confirm this demand.

Current options in equine rehabilitation training programs for undergraduate degree students are limited. For example, the University of Tennessee offers the only university-based, American Association of Veterinary State Boards Registry of Approved Continuing Education approved credentialed program in equine rehabilitation, but this is only available to veterinarians, veterinary technicians, physical therapists, or active students in these professions. Midway University offers an equine rehabilitation concentration within their Equine Studies Bachelor of Science program. This area of concentration includes similar courses to those described above, such as exercise physiology and equine rehabilitation courses focusing on understanding different therapeutic modalities and participating in hands-on experiences rehabilitating horses. Other programs offered by nonuniversity organizations often include considerable cost to the participant, as well as a need to travel to the location for the hands-on part of the training. The added cost and travel make this inaccessible for many students. Thus, creating a minor that integrates into the animal science curriculum allows students to gain additional knowledge and hands-on skills, better preparing them for careers in the horse industry.

## Supplementary Material

txad042_suppl_Supplementary_Data
