# Supplementary material for: Skills for future equine sports rehabilitation careers
Source: Transl Anim Sci. 2023 Apr 24;7(1):txad042. doi: 10.1093/tas/txad042 (PMC10199784; doi:10.1093/tas/txad042)
Supplement: txad042_suppl_Supplementary_Data [file txad042_suppl_supplementary_data.docx]

Supplement 1. Survey text.

Equine Rehabilitation Management

Q1.1 Principal Investigator: Sarah Reed
Title of Study: Assessing needs and interests to inform curriculum development in equine rehabilitation management

You are invited to participate in a research study. This form includes information about the study and contact information if you have any questions.

The field of equine sports medicine and rehabilitation is rapidly expanding and there are increasing opportunities for individuals (non-veterinarians) to operate equine rehabilitation facilities. However, throughout the United States, there are limited educational opportunities for undergraduate students to prepare for this career. The goal of the proposed study is to determine what practical skills and theoretical knowledge are deemed most useful for employment in this industry. In this survey, you will be asked your perceptions of skills and knowledge that are important for non-veterinary professionals in the field of equine rehabilitation. 

This study should take approximately 10 minutes of your time. Your participation will be anonymous, unless you choose to provide your contact information. If you provide your contact information, the researchers may contact you in the future to complete a recorded follow up interview. You are not required to provide contact information or participate in the follow up interview. If you choose to participate in the follow up interview, it should take approximately 30 minutes of your time. 

You will not be paid for being in this study. We do not anticipate any risks from completing the survey. You may not benefit from this research. However, the data collected are expected to inform curriculum design and therefore may benefit future students. 

Any data collected during this research study will be kept confidential by the researchers. Your survey responses will be kept on a secured server. The researchers will code the responses using a random six digit number and identifying information (if provided) will be removed. A master key that links the identifying information with codes will be maintained in a separate and secure location. Recordings from follow up interviews will be uploaded to a secure password-protected computer in the researcher’s office and will be coded in the same manner described above. The master key and the recordings will be stored for 3 years after the study has been completed and then destroyed. 

We will do our best to protect the confidentiality of the information we gather from you but we cannot guarantee 100% confidentiality. Your confidentiality will be maintained to the degree permitted by the technology used. Specifically, no guarantees can be made regarding the interception of data sent via the Internet by any third parties.” You do not have to be in this study if you do not want to be. 

You do not have to answer any question that you do not want to answer for any reason. We will be happy to answer any questions you have about this study. If you have further questions about this project or if you have a research-related problem, you may contact Sarah Reed at sarah.reed@uconn.edu. If you have any questions about your rights as a research participant, you may contact the University of Connecticut Institutional Review Board (IRB) at 860-486-8802. The IRB is a group of people who review research studies to protect the rights and welfare of research participants. 

Please print out a copy of this information sheet for your records. 

If you would like to participate in this survey, click yes to begin or no to exit.

- Yes
- No

Q1.2 In what country are you located?

▼ United States of America (USA) ... Zimbabwe

Q1.3 In what state are you located?

▼ Alabama ... Wyoming

Q1.4 What is your role in the equine rehabilitation industry?

- Veterinarian
- Veterinary Technician/Assistant
- Rehabilitation Farm Manager/Owner
- Rehabilitation Farm Staff
- Other (please describe) __________________________________________________

Q1.5 If you are a veterinarian, what is your primary specialty?

- Anatomic pathology
- Bacteriology/Mycology
- Cardiology
- Clinical pathology
- Dentistry
- Epidemiology
- Equine (general)
- Equine diagnostic imaging
- Equine Rehabilitation
- Immunology
- Large animal internal medicine
- Large animal surgery
- Neurology
- Oncology
- Parasitology
- Radiation oncology
- Shelter medicine
- Virology
- Other (please list) __________________________________________________

Q1.6 What practical skills do you believe are essential for professionals in the equine rehabilitation industry?

- Wrapping
- Ultrasound
- Massage
- Heat/cold therapies
- Basic handling
- Riding
- Communication
- Other (please list) __________________________________________________

Q1.7 Of the practical skills listed, which do you believe to be the most important?

- Wrapping
- Ultrasound
- Massage
- Heat/cold therapies
- Basic handling
- Riding
- Communication
- Other (please list) __________________________________________________

Q1.8 What medical knowledge do you believe are essential for professionals in the equine rehabilitation industry?

- Evaluation of lameness/biomechanics
- Anatomy
- Fundamentals of exercise reconditioning programs (for example, overload, progression, etc.)
- Other (please list) __________________________________________________

Q1.9 Of the medical knowledge listed above, which do you believe to be most important?

- Evaluation of lameness/biomechanics
- Anatomy
- Fundamentals of exercise reconditioning programs (for example, overload, progression, etc.)
- Other (please list) __________________________________________________

Q14 Are there specific skills or qualities you look for in employees that will be rehabilitating horses? If so, what are they?

Q1.10 Please provide any additional comments you feel may be helpful.

Q1.11 If you are willing to be contacted for a follow-up interview or would like to learn about the results of the study, please provide your name and email address.
